# Experiences from the pilot implementation of the Package of Essential Non-communicable Disease Interventions (PEN) in Myanmar, 2017-18: A mixed methods study

**DOI:** 10.1371/journal.pone.0229081

**Published:** 2020-02-18

**Authors:** Lwin Lwin Aye, Jaya Prasad Tripathy, Thae Maung Maung, Myo Minn Oo, Mya Lay Nwe, Hlaing Moh Moh Thu, Ko Ko, Kyaw Kan Kaung

**Affiliations:** 1 NCD Prevention and Control Division, Department of Public Health, Ministry of Health and Sports, Nay Pyi Taw, Myanmar; 2 International Union Against Tuberculosis and Lung Disease, The Union South East Asia Office, New Delhi, India; 3 International Union Against Tuberculosis and Lung Disease, Paris, France; 4 Department of Medical Research, Ministry of Health and Sports, Nay Pyi Taw, Myanmar; 5 International Union Against Tuberculosis and Lung Disease, Mandalay, Myanmar; 6 Department of Human Resources for Health, Ministry of Health and Sports, Yangon, Myanmar; Tabriz University of Medical Sciences, IR Iran, ISLAMIC REPUBLIC OF IRAN

## Abstract

**Background and objectives:**

Myanmar adopted the World Health Organization (WHO) Package for Essential Non-Communicable Disease Interventions (PEN) in 20 pilot townships in 2017. This study was conducted to assess the implementation of PEN, its effectiveness and understand the facilitators and barriers in its implementation.

**Methods:**

Mixed methods design involving a quantitative component (retrospective study analysing both aggregate and individual patient data from PEN project records; cross-sectional facility survey using a structured checklist) and a descriptive qualitative component.

**Results:**

A total of 152,446 individuals were screened between May 2017-December 2018 comprising of current smokers (17.5%), tobacco chewers (26.3%), Body Mass Index ≥25 kg/m^2^ (30.6%), raised blood pressure i.e. ≥ 140/90 mmHg (35.2%) and raised blood sugar i.e. Random Blood Sugar >200 mg/dl, Fasting Blood Sugar >126 mg/dl (17.1%). Nearly 14.8% of those screened had Cardiovascular Disease (CVD) risk score ≥20%, 34.6% had CVD risk not recorded. Of 663 patients registered with diabetes and/or hypertension in 05 townships, 27 (4.1%) patients made three follow-up visits after the baseline visit, of whom, CVD risk assessment, systolic blood pressure and blood sugar measurement was done in all visits in 89.0%, 100.0% and 78.0% of cases respectively. Health facility assessment showed 64% of the sanctioned posts were filled; 90% of those appointed been trained in PEN; key essential medicines for PEN were available in half of the facilities surveyed. Confidence of the health care staff in managing common NCD and perceived benefits of the project were some of the strengths.

**Conclusion:**

High loss to follow up, poor recording of CVD risk score, lack of essential medicines and equipments were the key challenges identified that need to be addressed before further expansion of PEN project to other townships.

## Introduction

Non-communicable diseases (NCDs) accounted for nearly 72% of all deaths in 2016 with three quarters of these deaths occurring in low- and middle-income countries (LMICs) [1]. While death rates have significantly declined in some developed countries, morbidity and mortality due to NCDs have increased at an alarming rate in the LMICs [2].

To curb the rising epidemic in the LMICs, the World Health Organization (WHO) developed a Package of Essential Non-Communicable (PEN) Disease Interventions for low-resource primary care settings. PEN is a set of cost-effective interventions, both population-wide and individual level, including health education, promotion of healthy behaviours, early diagnosis of NCDs and their risk factors. It employs inexpensive technologies, affordable medications for prevention and treatment of cardiovascular disease (CVD), stroke, diabetes, hypertension, cancer and asthma, regular follow-up and referral [3].

There is little global evidence about the implementation and effectiveness of PEN interventions in programmatic settings. Previous studies in the Democratic People’s Republic of Korea, Bhutan and Kyrgyzstan have yielded contrasting results in terms of its implementation and effect on CVD risk, blood pressure and blood sugar control [4,5]. Kontsevaya et al. suggested qualitative methods for a clearer understanding of the challenges and guide adjustment of the PEN intervention model [6].

Myanmar is experiencing a rapidly evolving epidemiological transition marked by an increase in the NCD burden whilst the prevalence of infectious diseases is still substantial [7]. NCDs accounted for 59% of deaths in Myanmar in 2014 [8]. A recent STEPS survey in Myanmar also showed a substantial burden of NCDs and its risk factors such as tobacco use, alcohol use, obesity, insufficient physical inactivity and 10-year CVD risk [9].

To tackle this growing threat, Myanmar adopted the WHO PEN interventions. Encouraged by the findings of the pilot PEN project in two townships of Yangon in 2012, the project was extended to 20 townships across five states/regions in March 2017 [10]. There has been no evaluation of this project in Myanmar. As Myanmar is planning to extend this intervention to all states/regions by 2019, it is a good time to reflect. This mixed-methods assessment of the PEN will provide the NCD Control Program with invaluable information on the performance of the project, utilization of health services by the community and the challenges faced during its implementation. This would also aid planning of program expansion to other townships.

Thus, the present operational research study was conducted with the aim of evaluating the feasibility, health system preparedness and effectiveness of the PEN interventions and to identify facilitators and barriers in implementing the interventions. The specific objectives of the study are:

1. Determine the number (proportion) of NCDs and its risk factors among those screened under PEN in 20 townships of Myanmar 2. Among those registered with diabetes and/or hypertension during May-Oct 2017 in 5 selected project townships, to determine the: a) number (proportion) with 0, 1, 2 or 3 follow-up visits in addition to the initial visit, b) number (proportion) who adhered to the follow-up patient monitoring protocol (CVD risk assessment, blood glucose, blood pressure measurement, eye and foot examination, counselling for tobacco cessation)among those who had at least 3 visits, c) number (proportion) who had reduction of CVD risk score, blood pressure and blood glucose in the latest visit compared to the initial visit among those who had at least 3 visits, 3) assess the preparedness of the health facilities in implementing the PEN interventions, and 4) understand the facilitators and barriers in implementing the PEN interventions from a health care providers’ perspective.

## Methods

### Study design

A sequential explanatory QUAN-QUAL mixed methods design [11] involving a quantitative component (retrospective study using aggregate PEN project data for objective 1 and individual patient level data for objective 2; cross-sectional facility survey using a structured checklist for objective 3) and a descriptive qualitative component. This is an evaluation of the implementation of PEN project with no comparison arm.

### Setting

#### General setting

Myanmar is a Southeast Asian country with 51 million inhabitants, 70.0% of whom reside in rural areas [12]. It is divided into seven states, seven regions and one capital territory (Nay Pyi Taw Council territory) which are further subdivided into 74 districts with 330 townships [13].

The curative health services are provided by the general/specialist hospitals, teaching hospitals, state/regional hospitals, district hospitals, township health facilities and station hospitals. In the rural areas, primary health care services are provided by the Rural Health Centres (RHCs). Each RHC is manned by the Basic Health Staff (BHS) which include one health assistant (HA), one lady health visitor (LHV), one public health supervisor-II (PHS-II) and two midwives (MWs). In the urban areas, Urban Health Centres (UHCs) and Maternal and Child Health Centres provide primary health care services. They are manned by medical officer(s) and other BHS. These BHS play a key role in the implementation of NCD prevention and control activities.

#### Specific setting: WHO PEN project in Myanmar

Initiation of the PEN project: In Myanmar, WHO PEN implementation began in 2012 in two townships, Hlegu and Hmawbi in Yangon Region initiated by the Diabetes Prevention and Control Project under the Department of Health. In 2017, following the success of the project, the pilot PEN project was expanded to 20 townships across five states/regions under the Non-communicable Disease Prevention and Control Division, Department of Public Health. WHO PEN protocols were adapted for Myanmar with the support of an technical expert group. Training of Trainers (TOT) was given to township medical officers and senior basic health staffs such as Township Health Assistant (THA) and Township Health Nurse (THN). At the township level, multiplier trainings were provided to local BHSs by township training team who received TOT. The essential resources such as BHS manual for NCD screening and treatment, diagnostic equipment such as BP cuff, Glucometer, Glucostrip and lipid analysers and essential drugs for PEN were provided in the pilot townships.

*Description of the intervention*. The PEN intervention aimed at improving the efficiency and quality of care for major NCDs in primary health care setting through the BHS at the UHC, MCH, RHC or the Sub-RHC level. All patients attending the outpatient clinics of RHCs/UHCs/MCH clinics in the pilot townships are assessed for eligibility criteria. Those who are over 40 years of age or obese or smoker or with family history of Diabetes/CVD/renal disease/hypertension are screened by the BHS. The patient details are recorded in the NCD Screening Register. The screening process includes 10-year CVD risk assessment using WHO CVD/ISH risk prediction charts, enquiry about tobacco use, alcohol use, symptoms of oral, breast and cervical cancers, measurement of weight, height, waist circumference and blood pressure [14]. These screening clinics are also held in remote far off locations on specific days, also known as mobile clinics.

**Fig 1:**
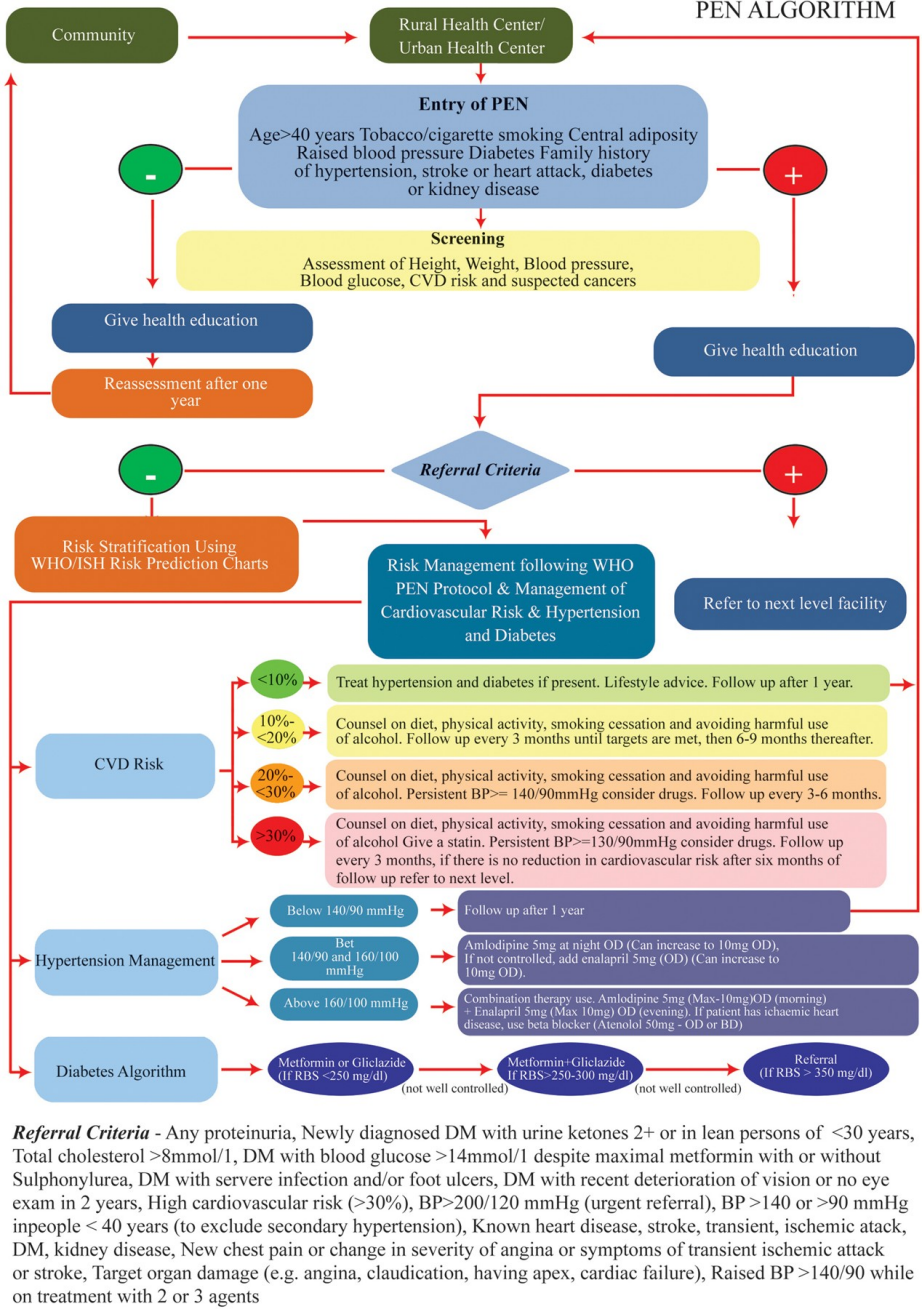
Screening, diagnosis, management and referral algorithm followed in the PEN project in Myanmar, 2017–18.

Cases of diabetes, hypertension (both newly diagnosed and already known) and suspected cancers are entered in the NCD Disease register. Cases of diabetes and hypertension requiring referral (using defined criteria given in Fig 1) and all those with suspected cancers are referred to the station and township hospitals for further management.

A clinical record form is filled in for those presenting with an NCD and for those who are found to have high blood pressure or high blood glucose during screening. For every outpatient visit, patients are asked whether they had been registered earlier; if so, the clinical record is retrieved. In every follow-up visit the following activities are done: CVD risk assessment, blood pressure measurement, blood glucose estimation, BMI estimation, counselling, enquiry about eye/foot examination and whether they have taken all medications regularly since their last visit. The BHS provide appropriate treatment to the patients according to the treatment guidelines outlined in the PEN protocol. They prescribe the following medicines: Metformin 500mg, Gliclazide80mg, Amlodipine 5mg, Enalapril 5mg, Atenolol 50mg, Atorvastatin 10mg and Aspirin 75mg[14]. These data are recorded in the clinical record form during every follow-up visit. Patients with 10-year CVD risk >10% are expected to visit every three months or more if needed. The details of the project are outlined in Fig 1.

Reporting: On the last week of each quarter, BHS use tally sheets to abstract data from the screening register, NCD disease register and clinical forms, to prepare a quarterly report that is sent to the township health facility. Reports from the township level are collected and compiled by the state/regional health departments and sent to Central NCD office in Nay Pyi Taw.

### Study period

The data collection for the study was carried out between November 2018 and May 2019.

### Sample size, sampling and study population

For objective 1, aggregate data on all patients screened for NCDs and its risk factors in 20 townships of Myanmar under the pilot PEN project during May 2017-Dec 2018 were analysed.

For objective 2, one township was selected randomly from each of the five states/regions. From each township (n = 5), two RHCs were drawn randomly (n = 10 RHCs). All persons with diabetes and/or hypertension with 10-year CVD risk≥10% in the selected RHCs registered during May-Oct 2017 were recruited.

For health facility assessment, two health facilities were randomly selected from each of the 10 project townships (n = 20) finally including 08 UHCs/MCHs and 12 RHCs.

For the qualitative component, BHS were purposively sampled to cover all four types of staffs (LHV, HA, MW and PHS-II) in 05 randomly selected health facilities (03 RHCs and 01 UHC, 01 MCH), one each from 05 project townships. These townships were also selected randomly (one from each project implementing state/region).

### Study outcomes

The study outcomes are broadly of two types: implementation outcomes (feasibility, facilitators and barriers and health system preparedness) and clinical outcomes (reduction of CVD risk score, blood pressure and blood glucose). The implementation outcomes were studied by a combination of quantitative and qualitative methods whereas clinical outcomes were assessed by review of patient records. The operational definitions of key terms used are given in Box 1.

**Box 1. Operational definitions of key terms used in the study.**

**10-year CVD risk:** It was calculated using WHO/ISH risk prediction charts and categorized into: <10%; 10-<20%; 20-<30% and ≥30%. There are two sets of charts. One set can be used in settings where blood cholesterol can be measured. The other set is for settings in which blood cholesterol cannot be measured. The risk calculation takes into account the following information: i) presence or absence of diabetes, ii) gender, iii) smoking status iv) age, v) systolic blood pressure, and vi) total blood cholesterol (if available)

**Diabetes mellitus:** Fasting Blood Glucose ≥ 126 mg / dL (7.0 mmol / L) (fasting was defined as no caloric intake for at least 8 hours) or 2—hour Post prandial ≥ 200 mg / dL (11.1 mmol / L) or HbA1C ≥ 6.5% (48 mmol/mol) or in a patient with classic symptoms of polyuria or polydipsia or weight loss or hyperglycemia or hyperglycemic crisis, a random plasma glucose ≥ 200 mg / dL (11.1 mmol / L) or known case of diabetes. In the absence of unequivocal hyperglycemia, results were confirmed by repeat testing.

**Hypertension (HTN):** Blood pressure ≥ 140/90 mmHg. (two BP readings 5 minutes apart in sitting position using Omron digital BP equipment) or known case of HTN.

**Reduction in CVD risk:** i) Any reduction in CVD risk level in the latest visit compared to the initial visit among those who had at least three visits.

**Adherence to monitoring protocol:** Among those with at least three visits, adherence was assessed for three key activities and defined as i) CVD risk assessment done in each visit, ii) blood pressure measurement done in each visit, iii) among persons with diabetes, blood glucose done at least once besides the initial assessment, iv) eye examination, foot examination and counselling for tobacco cessation done at each visit.

**Current smoking/chewing:** Any smoking/chewing in the last 30 days.

**Heavy alcoholic:** Drinking alcohol 4 days or more in a week **Obesity:** Body Mass Index ≥ 30 kg/m^2^.

**Overweight:** Body Mass Index ≥ 25 kg/m^2^.

### Data collection and source of data

#### Quantitative component

For objective 1, aggregate data (number of patients screened and number with various NCDs and their risk factors) were collected from the Quarterly NCD screening report that is available at the central NCD division at Nay Pyi Taw.

For the second objective, individual patient level data were obtained from patient clinical record forms which are maintained at the RHCs/UHCs by the BHS.

A structured health facility survey checklist was used to assess the status of manpower, trained staff, availability of medicines and equipment in 20 selected facilities.

#### Qualitative component

A total of 22 KIIs with four cadres of BHS were conducted. Participants were recruited until no new relevant information pertaining to the major themes was being obtained. The principal investigator (PI) conducted the interviews on a day and time convenient to the participants, preferably in a health facility. The PI is a National NCD Program staff in Myanmar (Female, Masters in Public Health) and is trained in qualitative research methods. Participants were informed of the purpose of the study before each KII. Interviews were done after obtaining their permission and consent to participate in the study. Only the participant and the researcher were present during the interviews. The interviews lasted for around 30–45 minutes. They were done in local language in which both the participant and the PI are familiar with. A pilot tested interview guide with broad open-ended questions was used to guide the interview. Audio recording (after consent) was done and verbatim notes were also taken during the KIIs.

### Data analysis and statistics

#### Quantitative

Data were double entered and validated in EpiData entry (EpiData association, Odense, Denmark) and analysed using EpiData analysis. Number and proportion were used to present key outcomes such as proportion of individuals with NCDs and their risk factors, pattern of follow-up visits, adherence to patient monitoring protocol and reduction in CVD risk. Logistic regression (ENTER method) was used to explore patient’s socio-demographic and clinical factors potentially associated with follow-up visit. Outcome variable was defined as any follow-up visit after the initial visit.

#### Qualitative

The KIIs were transcribed by the PI in *Burmese* language as soon as data collection was over. The transcripts were read several times to increase familiarity with the content. Manual descriptive content analysis was done by the PI and another investigator (TMM) to analyze the transcripts[11,15]. Codes were generated to depict the pattern of responses in the transcript. Similar codes were combined to form themes. A thematic framework was developed based on the themes emerging from the transcript. This was then reviewed by another investigator (TMM) to reduce subjectivity in interpretation of data and provide a different perspective to the participants’ responses. In case of any discrepancy, a third investigator was consulted (JPT). If required, the transcripts and the audio files were referred back. The findings have been reported using ‘Consolidated Criteria for Reporting Qualitative Research’(COREQ) guidelines[16]. Verbatim quotes have been presented in *italics* after translating into English.

### Ethical approval

Ethical approval for the study was obtained from the Ethical Review Committee (ERC) of the Department of Medical Research, Myanmar and the Union Ethics Advisory Group of the International Union of Tuberculosis and Lung disease (The Union), Paris, France. Administrative permission for the study was received from the NCD Unit, Myanmar.

## Results

### Socio-demographic, clinical and risk factor profile of participants

A total of 152,446 individuals were screened for NCD risk factors and common NCDs, of whom, 103,791 (68%) were females. Among those screened, the proportion of NCD risk populations such as current smokers, tobacco chewers and heavy alcoholics were 17.5% (n = 26,683), 26.3% (n = 40,023) and 5.3% (n = 7,925) respectively. Overall one-third (46,673, 30.6%) had BMI more than 25 kg/m^2^. More than one-third had hypertension (n = 53,616, 35.2%), with a large majority (45,435, 85%) being new cases. The prevalence of diabetes was found to be 17.1% (n = 26,092), predominantly new cases (22,322, 85%). Nearly 14.8% (n = 22,508) of those screened had CVD risk score >20%, 52,811 (34.6%) did not have their CVD risk score recorded. A total of 114 cases of oral cancer, 37 cases of breast and 10 cases of cervical cancer were screened positive. **(Table 1)**

**Table 1:** Results of screening of patients for common non-communicable diseases and their risk factors in 20 townships of Myanmar under the PEN project from May 2017-December 2018.

Variable	Male n (%)	Female n (%)	Total
Total screened	48,655	1,03,791	152,446
Current smokers	16,623(34.2)	10,060(9.7)	26,683 (17.5)
Current tobacco chewers	19,105(39.3)	20,918(20.2)	40,023 (26.3)
Heavy alcoholics*	7,025(14.4)	900(0.9)	7,925 (5.2)
BMI ≥ 25 kg /m^2^	13,983(28.7)	32,690(31.5)	46,673 (30.6)
Hypertension			
Known	2,682(5.5)	5,499(5.3)	8,181 (5.4)
New cases	15,831(32.5)	29,604(28.5)	45,435 (29.8)
Diabetes			
Known	1,148(2.4)	2,622(2.5)	3,770 (2.5)
New cases	5,652(11.6)	16,670(16.1)	22,322 (14.6)
CVD risk score			
<10%	13,971(28.7)	31,945(30.8)	45,916 (30.1)
10- <%	9,452(19.4)	21,759(21.0)	31,211 (20.5)
20-<30%	4,664(9.6)	9,678(9.3)	14,342 (9.4)
30-<40%	1,651(3.4)	2,856(2.8)	4,507 (3.0)
≥40%	1,112(2.3)	2,547(2.5)	3,659 (2.4)
Not recorded	17,805(36.6)	35,006(33.7)	52,811(34.6)
Suspected Cancer			
Oral	45(0.1)	69(0.1)	114 (0.1)
Breast	0(0)	37(0.0)	37 (0.0)
Cervix	-	10(0.0)	10 (0.0)
Referral to higher center	511(1.1)	2,994(2.9)	3,505(2.3)

BMI: Body Mass Index; CVD: Cardiovascular Disease.

PEN: Package of Essential Non communicable Disease Interventions.

* Drinking alcohol 4 days or more in a week ***Pattern of follow up visits***.

**Fig 2:**
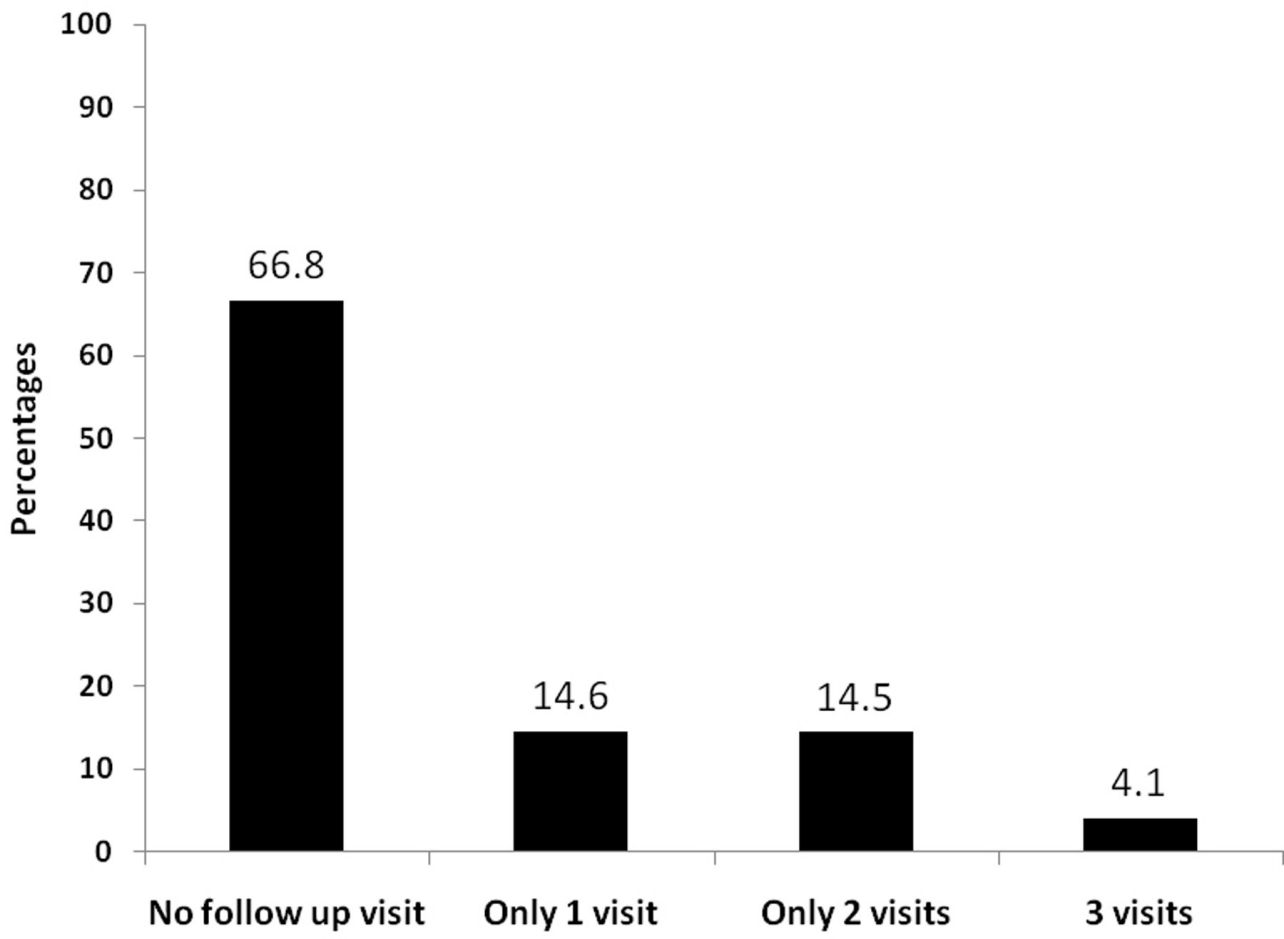
Pattern of follow up of patients diagnosed and registered with hypertension or diabetes or both in 5 selected townships implementing PEN project, Myanmar, between May- October 2017.

A total of 663 were diagnosed and registered with diabetes and/or hypertension in five selected townships between May-October 2017. Most of them were females (457, 69.0%) with a mean (SD) age of 60 (12.8) years. Nearly 28% (n = 186) had CVD risk score more than 20%. A total of 418 (63.1%) had hypertension, 112 (16.9%) had diabetes and 99 (14.9%) had both. (**Table 2)** A total of 27 (4.1%) patients made three follow-up visits after the baseline visit. **Fig 2**

**Table 2:** Characteristics of patients diagnosed and registered with hypertension or diabetes or both in 5 selected townships implementing PEN project, Myanmar, between May- October 2017.

Variable	N	(%)
**Total**	663	(100)
**Mean age in years (SD)**	60	(12.8)^
**Age group (in years)**		
< = 40 yrs	46	(6.9)
41–60 yrs	287	(43.3)
> = 60 yrs	325	(49.0)
Not recorded	05	(0.8)
**Sex**		
Male	176	(26.5)
Female	457	(69.0)
Not recorded	30	(4.5)
**10 year CVD Risk**		
< 10%	282	(42.5)
10 –<20%	171	(25.8)
20 –<30%	103	(15.5)
30 –<40%	28	(4.2)
≥ 40%	55	(8.3)
Not recorded	24	(3.6)
**RBS** (Mean, SD) (n = 524) in mg/dl	173	(91)^
**FBS** (Mean, SD) (n = 117) in mg/dl	136	(56)^
**Systolic Blood Pressure** (Mean, SD) n = 657 in mm of Hg	143	(22)^
**Diastolic Blood Pressure** (Mean, SD) n = 623 in mm of Hg	89	11^
**Use of tobacco products**		
Smoking	84	(12.7)
Smokeless tobacco	149	(22.5)
**Type of disease**		
Diabetes	112	(16.9)
Hypertension	418	(63.1)
Both	99	(14.9)
Not recorded	34	(5.1)

^figures in this column represent mean and standard deviation (SD).

FBS: Fasting Blood Glucose; RBS: Random Blood Glucose;

CVD: Cardiovascular Disease; PEN: Package of Essential Non communicable Disease Interventions.

### Adherence to follow-up protocol and assessment of clinical outcomes

Among those with three follow-up visits, CVD risk assessment, systolic blood pressure and blood sugar measurement was done in all visits in 89.0%, 100.0% and 78.0% of cases respectively. Reduction (baseline visit versus third follow-up visit) of CVD risk score, blood sugar and systolic blood pressure was seen in 26.0%, 60.0% and 31.0% of cases. (**Table 3)**

**Table 3:** Adherence* to follow up protocol and control/reduction of risk factors in the third follow up visit compared to the first visit among those with at least 3 follow up visits (n = 27).

Adherence to follow-up care protocol	N	(%)
CVD risk assessment	24	(89)
BMI assessment	18	(67)
Referral for eye examination among patients with diabetes (n = 20)	4	(15)
Measurement of blood pressure (SBP)	27	(100)
Measurement of blood pressure (DBP)	24	(89)
Blood sugar measurement at least once	24	(89)
Blood sugar measurement in all three follow-up visits	21	(78)
Referral for foot examination among patients with diabetes (n = 20)	18	(65)
Counselling for tobacco use and cessation	13	(48)
**Control/reduction of risk factors**		
CVD risk score		
• Reduction	7	(26)
• No change	14	(52)
• Raised	4	(15)
• Could not be assessed	2	(07)
Blood sugar		
• Reduction	16	(60)
• Raised	5	(18)
• Could not be assessed	6	(22)
Systolic blood pressure		
• Reduction	15	(55)
• No change	4	(15)
• Raised	8	(30)
Diastolic blood pressure		
• Reduction	8	(31)
• No change	9	(33)
• Raised	7	(26)
• Could not be assessed	3	(11)

SBP: Systolic Blood Pressure; DBP: Diastolic Blood Pressure.

CVD: Cardiovascular Disease; BMI: Body Mass Index.

*Adherence is defined as assessment done in all three follow-up visits.

### Health system preparedness

Twenty health facilities were surveyed for preparedness including 8 UHCs/MCHs and 12 RHCs. Regarding human resource availability, overall, 64.0% of the sanctioned posts was filled; 34.0% and 83.0% of sanctioned posts for PHS-II and midwife posts were filled. Nearly 90.0% of those appointed have been trained in PEN. **(Table 4)** Majority of the equipments (>80.0%) were functional, except for digital BP machine (73.0%). **(Table 5)**

**Table 4:** Availability of human resource at 20 PEN implementing facilities in Myanmar in 2017–18.

Health facility resources	Number sanctioned	Number appointed (%^μ^)	Number trained (%*)
**Human resource**			
Health Assistant	11	11 (100)	11 (100)
Lady Health Visitor	20	18 (90)	17 (94)
Midwife	64	53 (83)	47 (89)
PHS-II	71	24 (34)	19 (79)
**Overall**	**166**	**106 (64)**	**94 (89)**

PEN: Package of Essential Non Communicable Disease Interventions.

PHS: Public Health Supervisor.

^μ^ This percentage is calculated as: (number appointed/number sanctioned)*100.

*This percentage is calculated as: (number trained for PEN/number appointed)*100.

**Table 5:** Availability of functional equipments at 20 PEN implementing facilities in Myanmar in 2017–18.

Equipments	Total N	Functioning n (%)^¥^
Digital BP cuff	49	36 (73)
Weighing machine	41	37 (90)
Measuring tape*	44	44 (100)
Stethoscope	54	43 (80)
Glucometer	70	57 (81)
**Overall**	**258**	**217**

^¥^This percentage is calculated as: (number of functional equipments/total equipments)*100.

PEN: Package of Essential Non Communicable Disease Interventions.

WHO CVD risk chart, BMI chart and NCD manual for BHS were available in all the health facilities, amlodipine and atenolol was found in three-fourth of the facilities, whereas other drugs such as Metformin, Gliclazide and atorvastatin was seen in about half of the health facilities surveyed.**(Table 6)**.

**Table 6:** Availability of medicines and other supplies at 20 PEN implementing facilities in Myanmar in 2017–18.

Availability of medicines/equipments	Total facilities surveyed	N (%)
NCD BHS manual	20	20 (100)
BMI Chart	20	20 (100)
WHO CVD Risk chart	20	20 (100)
Metformin	20	11 (55)
Gliclazide	20	10 (50)
Amlodipine	20	15 (75)
Atenolol	20	15 (75)
Enalapril	20	13 (65)
Aspirin	20	13 (65)
Atorvastatin	20	10 (50)

PEN: Package of Essential Non Communicable Disease Interventions.

NCD: Non communicable disease; BHS: Basic Health Staff; WHO: World Health Organization; CVD: Cardiovascular Disease;.

### Factors associated with LTFU

Female sex (aOR: 1.5, 1.1–2.4), younger age group <40 years (aOR: 1.4, 1.2–2.9) and being overweight (aOR: 4.3, 2.3–6.7) or obese (aOR: 3.1, 1.7–5.9) were significantly associated with LTFU. Individuals with both DM and HTN (aOR: 0.3, 0.2–0.7) had a significantly lesser odds of being LTFU compared to those with DM alone. **(Table 7)**

**Table 7:** Factors associated with loss to follow up among patients registered at PEN implementing facilities in Myanmar in 2017–18 (N = 540).

Factors	Unadjusted OR (95% CI)	Adjusted OR (95% CI)	p-value
**Female sex**	1.4 (1.0–2.2)	1.5 (1.1–2.4)	0.03
**Age categories**			
<40 years	1.5 (1.3–3.0)	1.4 (1.2–2.9)	0.01
41–60 years	1.0 (0.7–1.5)	1.1 (0.7–1.7)	0.6
>60 years	1.0	1.0	-
**Body Mass Index**			
Normal/Underweight	1.0	1.0	-
Overweight	4.2 (2.4–6.0)	4.3 (2.3–6.7)	<0.001
Obesity	3.0 (1.7–5.7)	3.1 (1.7–5.9)	<0.001
**CVD risk score**			
<20% risk	0.7 (0.4–1.3)	0.7 (0.5–1.2)	0.2
>20% risk	1.0	1.0	-
**Type of NCD**			
Diabetes only	1.0	1.0	-
Hypertension only	1.9 (0.9–3.2)	1.9 (1.0–3.2)	0.06
Both	0.3 (0.2–0.8)	0.3 (0.2–0.7)	0.001

### Qualitative results

A total of 22 BHS were interviewed (04 HA, 05 LHV, 07 MW and 06 PHS-II); majority were females (n = 19) and belonged to the age group of 15–44 years (n = 13). Nearly half of them had more than 10 years of total service (n = 10).

Two major themes were explored in the qualitative part: i) challenges and ii) facilitators in PEN implementation.

#### Programmatic challenges in PEN implementation

*Training related*. Some of the BHS said that the training they underwent for PEN was well delivered enabling them to carry out the project activities efficiently. However, they wanted refresher training once a year. Some of the staffs were not trained at all, especially the newly recruited ones which limits their role in project implementation.

*“One time training is not enough…..we need a refresher training to be able to perform better” -* 6 year service, Midwife.

They also pointed out some inconsistencies in training especially related to filling up of reports and management of diseases

A senior BHS said, “*sometimes there are discrepancies between what we had learnt in training and real practice…*.*we have to keeping asking for help from the township medical officer*”– 8 year service, male Health Assistant.

*Lack of human resources*. Most of the BHS from RHCs informed that they had enough manpower for implementation of PEN. But BHS from the urban areas such as UHCs and MCH centers said that that they are already overburdened due to the routine activities, additional responsibilities, relieving duties and field work which affected PEN clinical services.

“*Sometimes due to lack of adequate staff*, *the clinic opening hours gets extended by few hours…patients have to wait longer*”– 5 year service, Midwife.

*Challenges in the management of hypertension*. In most health centers, BHS gave anti-hypertensive drugs to patients for two weeks only due to insufficient supply of drugs. Frequent stock-out was common. So they had to ask the patient to buy medicines from private pharmacies which affects patient’s compliance to the drug.

Lack of functional medical equipment in some health facilities such as digital blood pressure cuff was another challenge.

*“Digital BP cuffs become dysfunctional after some period ……availability of other type of BP cuffs are also limited in RHC” -19 year service, LHV*.

*Challenges in the management of DM*. Shortage of drugs such as metformin and atorvastatin and supplies such as glucose strips and lancets were the key constraints in the management of diabetes. Some of them also expressed the need for additional training in management of diabetes as they seemed less confident about managing them. They also expressed difficulty in using lipid analyzers.

“We got three glucometers, but lancets were not sufficient because we did not get lancets last time. We gave back lipid analyzer as we did not know how to use it.

-19 year service, male Health Assistant.

*Challenges in calculating the CVD risk score*. Calculating the CVD risk score was not difficult for most of the BHS, except few who did not receive any training. The main challenge was referring patients with higher CVD risk score to a hospital because most of them were healthy and did not feel the need to go to a hospital.

Another challenge was calculating CVD risk score in mobile clinics or MCH clinics due to time constraints and lack of dedicated space for the clinic. Sometimes, due to lack of complete information, the risk score is not calculated.

“*We can do it…*. *but we cannot calculate the CVD risk score during mobile clinics*. *We have to do it when we are back home……so we are not able to inform the patient about their CVD risk*”– 6 year service, Midwife.

Referral issues.

Some staff reported, *“Although we refer patients to the hospital*, *we do not receive feedback from the referral hospital” -* 32 year service, LHV.

The staff also complained that most patients do not comply to referral instructions, because they are apparently healthy and don’t feel the need to visit a health facility.

*Recording and reporting*. The health staff stated that recording and reporting of forms in mobile clinics was difficult and time consuming compared to fixed clinics due to duplication of work.

“*On mobile clinic days*, *we register patient details in a separate mobile clinic register book and later extract NCD related data and fill the NCD register after returning to the health facility*”- 19 year service, LHV.

The staff complained that number of registers and forms were too many to be filled. The inconsistent information given by trainers for filling those forms also made their task difficult. Moreover, almost all health centers had to copy those registers and forms because of lack of supply of enough forms.

*Other operational challenges*. A BHS from an MCH center reported that their health center was located in the compound of a hospital, that’s why no NCD patient came to their health center at all for NCD screening. Also, this being an MCH center, opening the clinic during the daytime kept the people in the productive age group, especially males out of the facility.

#### Facilitators

*Perceived benefits of the program*. All the BHS felt that the current PEN package towards managing NCDs is important because it tackles a growing public health issue and should be continued and further expanded to other townships.

A senior LHV said emphatically *“If we can expand this project to all the townships in the country*, *NCD risk will go down”*

“Previously diagnosis and management of diabetes was done by doctors only, now patients can get early diagnosis and treatment for diabetes at the RHC”

– 20 year service, LHV.

Patients were happy to receive services such as blood sugar measurement, blood pressure measurement and medications for common NCDs free of cost. They appreciated the regular follow up service from the BHS and hoped that this service continues.

*Management of DM and hypertension*. The BHS were confident in managing cases of hypertension and DM as per the guidelines. If they needed some clarity, they took guidance from the Township Medical Officer over phone on a case-to-case basis.

*Availability of PEN manuals as a guide*. Most of the trained BHS told that they had PEN manuals. They also liked the content of the manual and felt that it was complete and a good reference book for practicing PEN services in clinic.

A senior LHV even went on to say that, *“I have not received the training…..but I give treatment and health education by reading the manual only and sometimes I consult the supervisors”*

## Discussion

This assessment of the PEN project in 20 townships of Myanmar yielded interesting findings and revealed key operational challenges to guide further expansion of the project. The six key findings of the study are: i) high prevalence of common NCDs and their risk factors, ii) high LTFU was observed with only 4% making three follow up visits after the initial visit, iii) female sex, younger age group, high BMI and having both DM and HTN were factors associated with LTFU, iv) CVD assessment was not done or not recorded in more than one-third of cases, v) among those who completed three follow up visits, CVD risk score reduction was seen in about one-fourth, vi) lack of trained human resource, recording and reporting issues, challenges in CVD risk score calculation and challenges in the management of NCD and DM were the key barriers.

First, an alarming proportion of the screened population was found to have high prevalence of common NCDs such as diabetes and hypertension and the risk factors of NCDs such as tobacco use, alcohol use and obesity. A community based nationwide STEPS survey in 2014 also revealed high prevalence of key NCD risk factors in the general population, although the figures were different.[9] This difference could be attributed to two reasons, i) difference in the nature of the sample, one being a community based sample and the other being a hospital based sample (primary care level), ii) varying definitions used for tobacco and alcohol use. Despite these differences, it is beyond any doubt that that there is a clear rising trend of NCDs and its risk factors in the country, mirroring the global shift from communicable to non-communicable diseases. This implies that the country should now be prepared for tackling the dual burden of diseases. The threat that CVD and other non-communicable diseases pose is now widely recognized with the World Health Assembly’s adoption of Global Action Plan for Prevention and Control of Non-communicable Diseases 2013–2020[17]. Myanmar should now strive to achieve the global “25 by 25” goal–achieving 25% relative reduction in overall mortality due to common NCDs by 2025 as outlined in the global action plan.

Second, although adherence to PEN protocol was good with modest reduction in CVD risk score, it has to be interpreted with caution due to high LTFU rates. Similar assessments of PEN elsewhere have yielded contrasting results in terms of its clinical effectiveness[4–6,18].

Third, a significant proportion of registered patients were lost to follow up after their initial visit. This is a matter of concern and needs to be urgently tackled before expanding the project to other townships because regular follow up and care is the hallmark of management of NCDs under this project. Regular follow up is key to achieving treatment goals, ensuring medication adherence and identifying complications at an earlier stage. The possible reasons for high LTFU in this study are: i) patients registered in mobile clinics were more likely to be LTFU because these clinics are conducted in far off remote locations with no health infrastructure which also makes it difficult for the BHS to trace back, ii) PEN clinics in MCH centers which are co-located within hospital premises also have higher LTFU rates because patients tend to visit the co-located hospital OPD for follow up care, iii) patients who are apparently healthy do not feel the need to visit hospital regularly, iv) other possible reasons include the time and costs associated with travel and the opportunity costs associated with it. PEN implementation in Bhutan showed high rates of follow-up due to the active involvement of the health workers who conducted home visits [5]. The study showed that female sex and patients in the younger age group were more likely to be lost to follow up, thereby requiring closer monitoring of these population groups. Patients with both DM and HTN were relatively more adherent to the follow up schedule compared to those having DM or HTN alone probably due to the perceived of their problem. We need rigorous qualitative research to explore the personal and health system related barriers to regular follow up from a patients’ perspective.

Fourth, CVD assessment was not done or not recorded in more than one-third of the instances despite the fact that the staff reported confidence in using the risk assessment chart. Possible reasons are: i) time and space constraints in mobile clinics and once a week clinics in the health facility rendered assessment of CVD risk score for every patient difficult as reported by the BHS, ii) in the mobile clinics the BHS capture requisite information for CVD risk calculation in the mobile clinic register, but fail to calculate the CVD risk score once they go back to the health facility due to other pressing priorities, iii) in some instances incomplete information rules out CVD risk score calculation, iv) poor recording could also be due to lack of supportive supervision and accountability. A study from similar settings in rural India demonstrated the feasibility of using a mobile application (SMART*health* India) in assessing CVD risk by village level health workers[19].

Fifth, health facility assessment found lack of adequate human resource in facilities implementing PEN project. This also emerged as one of the challenges in the qualitative part of the study. A similar nation-wide Service Availability and Readiness Assessment (SARA) in Myanmar in 2015 highlighted lack of trained staff at various levels of care.[20] This happens to be a common finding across studies in Africa and India[21–26].

Sixth, essential medicines for NCD management such as Metformin, Amlodipine, Gliclazide and Atorvastatin were found to be wanting with frequent stock outs. Qualitative interviews with providers also revealed interrupted supply of drugs as one of the major hindrance to the appropriate management of patients with DM and hypertension. This is supported by findings from other countries in similar settings[21,22,24–27]. Non-availability of medicines forces patients to purchase medicines from the private sector or forego treatment if they cannot afford it. It is essential to equip these facilities with regular supply of drugs before expansion of the pilot PEN project to other townships. Besides, lack of reporting forms and registers were also reported by the BHS as an operational constraint.

Seventh, it was observed during the review of the PEN implementation that screening, diagnosis and management of chronic respiratory diseases was sub-optimally or not implemented at all due to lack of adequate competency or confidence in performing the tasks.

Eighth, despite these implementation issues, it was noticed during interactions with the BHS that they were appreciative of this program and seemed confident enough to manage these new roles, which is quite encouraging and augurs well for the program. The strength of the program was the implementation in real-life conditions through non-physician health workers.

The study had some strengths. This is the first systematic evaluation of the PEN project in Myanmar and one of very few such studies across the globe. The quantitative and the qualitative findings corroborated well lending more validity to the study results. We adhered to the STROBE and COREQ guidelines for reporting the findings[16,28].

There were few limitations in this study. First, a large majority of the patients did not follow-up after registration which limits our interpretation of the clinical effectiveness of the PEN project. Second, we could not explore the reasons for poor follow-up rates from a patient’s perspective which could be an area for future research. Third, as the interviewer was a staff from the NCD program, the health staff may have been reluctant to criticise the programme, thus creating a degree of bias in their responses. Fourth, there was no comparison arm to strengthen the causal implication of the intervention’s impact.

### Policy implications

1. As CVD risk assessment is the cornerstone of this intervention package, more efforts should be directed towards ensuring completeness and correctness of this assessment through regular refresher trainings and on-field supportive supervision. Use of mobile application to calculate CVD risk score which has been tried successfully in similar settings could be tried[19].
2. Uninterrupted supply of drugs, equipments and other supplies should be ensured before expansion of the project.
3. Considering the difficulty in conducting clinics in mobile locations and high LTFU among patients registered in the mobile clinics, we recommend a re-consideration of this strategy or reformulation of this strategy with more meticulous planning and execution.
4. PEN clinics in MCH centres co-located within hospital premise have high LTFU rates which require better coordination between the two health facilities.
5. High LTFU is alarming and needs to be addressed through home visits or innovative use of technology. More research is required to identify the gaps and plug the losses.

## Conclusions

The PEN pilot project empowered non-physician primary health-care workers to extend screening, diagnostic, treatment and counselling services to patients with NCD from a health facility close to their home. This systematic evaluation identified several key challenges and strengths in the implementation of PEN in Myanmar which needs to be addressed before expansion of the project to other townships. Challenges in the calculation of CVD risk score, lack of medicines and high loss to follow up were the major drawbacks warranting urgent attention.

## Supporting information

S1 File List of abbreviation. (DOC)

S2 File Key informant interview/in-depth interview for provider side: Interview guide. (DOCX)
