# Supplementary material for: Experiences from the pilot implementation of the Package of Essential Non-communicable Disease Interventions (PEN) in Myanmar, 2017-18: A mixed methods study
Source: PLoS One. 2020 Feb 18;15(2):e0229081. doi: 10.1371/journal.pone.0229081 (PMC7028297; doi:10.1371/journal.pone.0229081)
Supplement: S1 File — (DOC) [file pone.0229081.s001.doc]

### List of abbreviation

| | BHS | Basic Health Staff | | --- | --- | | BMI | Body Mass Index | | BP | Blood Pressure | | COREQ | Consolidated Criteria for Reporting Qualitative Research | | CVD | Cardiovascular Disease | | FBS | Fasting Blood Sugar | | HA | Health Assistant | | ISH | International Society of Hypertension | | KII | Key Informant Interview | | LHV | Lady Health Visitor | | LMICS | Low- and middle-income countries | | LTFU | Loss to Follow Up | | MCH | Maternal and Child Health Center | | MW | Midwife | | NCD | Non-communicable Disease | | OPD | Out-Patient Department | | PEN | Package of Essential NCD Interventions | | PHC | Primary Health Care Center | | PHS-II | Public Health Supervisor Grade II | | QUAN-QUAL | Quantitative-Qualitative | | RBS | Random Blood Sugar | | RHC | Rural Health Center | | SARA | Service Availability and Readiness Assessment | | UHC | Urban Health Center | | WHO | World Health Organization | |  |
| --- | --- | --- | --- | --- | --- | --- | --- | --- | --- | --- | --- | --- | --- | --- | --- | --- | --- | --- | --- | --- | --- | --- | --- | --- | --- | --- | --- | --- | --- | --- | --- | --- | --- | --- | --- | --- | --- | --- | --- | --- | --- | --- | --- | --- | --- | --- | --- | --- | --- | --- | --- |
