# Supplementary material for: Experiences from the pilot implementation of the Package of Essential Non-communicable Disease Interventions (PEN) in Myanmar, 2017-18: A mixed methods study
Source: PLoS One. 2020 Feb 18;15(2):e0229081. doi: 10.1371/journal.pone.0229081 (PMC7028297; doi:10.1371/journal.pone.0229081)
Supplement: S2 File — (DOCX) [file pone.0229081.s002.docx]

**Assessment of implementation of the Package of Essential Non-communicable Disease Interventions (PEN) in Myanmar: a mixed methods study**

**Key Informant Interview/In-Depth Interview for provider side: Interview Guide**

Name of the participant:

Designation:

Date of Interview:

Interview start / end time:

Name of the Interviewer:

After a brief introduction to the participant regarding the purpose of the interview, the principal investigator will take informed written consent for the interview. Written informed consent will also be requested for audio recording.

1. Welcome

Introduce yourself and the notetaker

*Start with the following:*

- Who we are and what we’re trying to do
- What will be done with this information
- Why we asked you to participate
- Information provided will be kept confidential
- Read out information sheet and take written consent

*Topics and probes*

- Ask about the PEN implementation in the health facility
- Do they feel it is an important intervention and should it be continued?
- Programmatic challenges in its implementation
- Availability of equipments, drugs
- Adequacy of the training and training needs assessment
- Limitations in resources: manpower, money, time
- How do the community perceive about the services they receive
- Challenges in ensuring regular follow-up
- Challenges in assessing 10-year CVD risk, blood pressure measurement, blood glucose estimation
- Challenges in diet and tobacco counselling, foot examination
- Challenges in management and referral of diabetes and hypertension
- Challenges in reporting
- Suggestions to improve the implementation of the program
- Way forward

That concludes our interview. Thank you so much for coming and sharing your thoughts and opinions with us.
